# Prostaglandin D_2_ Synthase: A Novel Player in the Pathological Signaling Mechanism of the Aldosterone–Mineralocorticoid Receptor Pathway in the Heart

**DOI:** 10.3390/cells14191485

**Published:** 2025-09-23

**Authors:** Ankita Garg, Malte Juchem, Sinje Biss, Carla Nunes Borisch, Julia Leonardy, Christian Bär, Shashi Kumar Gupta, Thomas Thum

**Affiliations:** 1 Institute of Molecular and Translational Therapeutic Strategies (IMTTS), Hannover Medical School, 30625 Hannover, Germany; 2 Fraunhofer Institute for Toxicology and Experimental Medicine (ITEM), 30625 Hannover, Germany; 3 Fraunhofer Cluster of Excellence Immune-Mediated Diseases (CIMD), 30625 Hannover, Germany

**Keywords:** Aldo–MR pathway, cardiovascular disease, remodeling, cardiac hypertrophy, adeno-associated virus

## Abstract

**Background:** A deregulated aldosterone (Aldo)–mineralocorticoid receptor (MR) pathway is linked to cardiovascular disease (CVD), including hypertension and heart failure. Despite the association of elevated plasma Aldo levels with cardiac stress, inflammation, myocardial fibrosis, and cardiac remodeling, the underlying mechanisms remain elusive. **Methods:** To study the impact of Aldo–MR pathway overactivation on cardiac health, a novel mouse model with AAV9-mediated MR overexpression and Aldo administration via subcutaneous osmotic pumps was generated. Echocardiographic analyses, transcriptome sequencing, and loss-of-function experiments of an identified lead candidate gene were performed. Additionally, cardiac tissue samples from human patients with end-stage heart failure were analyzed in the study. **Results:** Mice with an overactivated Aldo–MR pathway exhibited increased neutrophil gelatinase-associated lipocalin (NGAL) expression, cardiac dysfunction, hypertrophy, and fibrosis. Transcriptomics identified prostaglandin D_2_ synthase (*Ptgds*) as a novel downstream effector of the cardiac Aldo–MR pathway. SiRNA-mediated inhibition of *Ptgds* in primary cardiomyocytes reduced NGAL levels and the hypertrophic impact of Aldo, suggesting a role in mediating Aldo-induced cardiac pathologies. Elevated expression of *PTGDS* was observed in hiPSC-CMs treated with the pro-hypertrophic cytokine leukemia inhibitory factor (LIF) and in end-stage heart failure patients, ascertaining its importance in cardiac disease settings. **Conclusions:** PTGDS is a newly identified mediator of Aldo–MR-induced cardiac remodeling and may represent a potential therapeutic target for CVD.

## 1. Introduction

Heart failure is a chronic clinical syndrome characterized by the inability of the heart muscle to pump sufficient blood throughout the body. This incapacity can originate from various reasons such as atherosclerosis, high blood pressure, cardiomyopathy, arrhythmias, or congenital heart disease. Cardiovascular diseases (CVDs) refer to conditions that affect the blood vessels or the heart and represent the leading cause of deaths and morbidity across the world, emanating an aggressive socio-economic burden on humanity and a substantial challenge for the scientific community [1].

Several studies advocate the role of a deregulated aldosterone–mineralocorticoid receptor (Aldo–MR) pathway in driving these cardiovascular pathologies, indicating the high importance of this pathway [2]. Under normal physiological conditions, the Aldo–MR pathway regulates the Na^+^-H_2_O balance in the body via kidneys’ ductal tubes, thereby maintaining the blood pressure [3,4]. However, an over-activated Aldo–MR pathway has been involved in myocardial infarction (MI), hypertension, and cardiac remodeling, including cardiac fibrosis, apoptosis, hypertrophy, inflammation, and oxidative cardiac stress, eventually leading to heart failure [5,6,7,8,9]. The therapeutic value of the Aldo–MR pathway has been explored using various MR antagonists that improved cardiac functions as observed in the clinical trials RALES, EPHESUS, and EMPHASIS-HF [10,11,12,13]. In addition to the studies showing an inverse correlation of activation and blockage of MR with cardiac health, inhibition of NGAL (downstream effector of Aldo–MR pathway) in mice was also found to limit cardiac damage induced by MI and ischemia-reperfusion injury [14,15,16,17]. Although a crucial role of the Aldo–MR pathway in mediating cardiac pathologies is evident, the detailed underlying mechanisms are still poorly understood.

**Figure 1:**
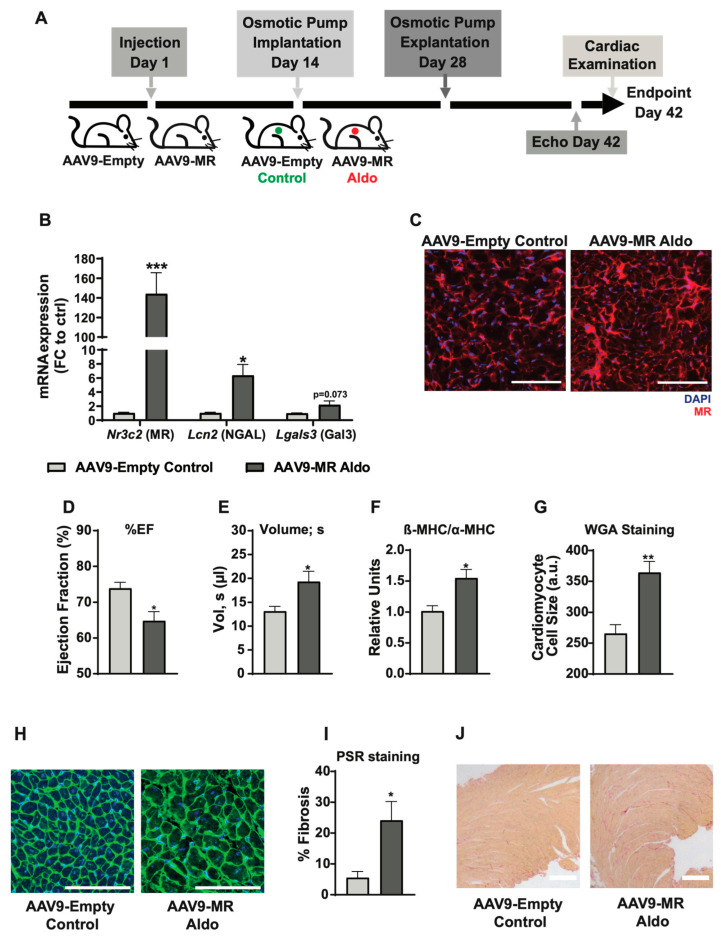
Aldo–MR over-activation mouse model. (**A**) A schematic overview of the experimental design for AAV9-mediated MR-overexpression in a murine model with pharmacological aldosterone treatment. (**B**) mRNA expression of various genes in mouse hearts treated with AAV9-Empty-Control and AAV9-MR-Aldo as measured by RT-PCR. (**C**) Fluorescent images obtained after Anti-MR staining of mouse heart cryo-sections from AAV9-Empty-Control and AAV9-MR-Aldo animal groups (*n* = 5–6 animals per group). (**D**,**E**) Echocardiographic analysis in the AAV9-mediated MR-overexpression system showing a change in ejection fraction percentage (**D**) and end-systolic volume (**E**) as a measure of cardiac function in mice with AAV9-Empty-Control and AAV9-MR-Aldosterone treatment (*n* = 5–6 animals per group). (**F**) Ratio of mRNA expression of β-MHC and α-MHC as analyzed by RT-PCR in mice with AAV9-Empty-Control and AAV9-MR-Aldosterone treatment (*n* = 5–6 animals per group). (**G**–**J**) Cell size measurement from heart tissue slices (**G**) after WGA staining (**H**), and percent of fibrosis (**I**) analyzed after Picro-Sirius red staining (**J**) in mice with AAV9-Empty-Control and AAV9-MR-Aldosterone treatment (*n* = 5–6 animals per group). All data are represented as mean ± SEM. * *p* ≤ 0.05, ** *p* ≤ 0.01, *** *p* ≤ 0.001. Scale bars indicate 100 (**C**,**H**) or 200 (**J**) μm.

To have an in-depth understanding of these cardiac pathologies, researchers have devised several small murine models such as coronary artery ligation (myocardial damage), transient occlusion of coronary artery (ischemia reperfusion damage), cryo-infarction, aortic constriction (pressure overload), and various transgenic models (e.g., mutation of α-actin protein for dilated cardiomyopathy) [18]. These physiological models hold great importance as they closely mimic cardiac pathological conditions and provide valuable mechanistic insight. Several knockout and transgenic models such as LDLR−/− mice (for familial hypercholesterolemia) and ApoE−/− mice (for atherosclerosis) are also available for exploring cellular pathways and evaluation of novel therapies [18]. Interestingly, a transgenic model for aldosterone-mediated cardiac hypertrophy was also developed by specific overexpression of 11β-hydroxysteroid dehydrogenase type 2 in cardiomyocytes that showed aggravated hypertrophy which improved upon treatment with Aldo antagonist eplerenone [19]. However, these models mainly focus on the genetic predisposition of cardiac pathologies, while the involvement of the Aldo–MR pathway in cardiac conditions is mostly acquired with age in adulthood rather than being genetic. Therefore, we developed a mouse model (adult mice, age 8–10 weeks) for Aldo–MR pathway activation where MR was overexpressed via AAV9 followed by subcutaneous implantation of osmotic pump implants for constant Aldo administration (Figure 1A). This model closely mimics the acquired Aldo–MR pathway over-activation in adulthood in mice without any predisposed cardiac conditions since birth. Here, using this mouse model, we identified key players involved in the Aldo–MR pathway-mediated cardiac remodeling and their functionalities.

## 2. Materials and Methods

### 2.1. Animal Model

For this study, 8–10-week-old male C57BL/6N mice were used. The sample size was determined by the original exploratory design of the study (*n* = 5 for control and *n* = 6 for overexpression group). On day 1 of the experimental timeline, AAV9-Empty/AAV9-MR was injected (i.v.) in the animals at a concentration of 2 × 10^12^ viral particles per animal (injection volume: 100 µL). After 14 days of AAV injection, osmotic pumps (Alzet^®^ model; ALZET LLC, Campbell, CA, USA) with either saline (0.9% NaCl) or aldosterone (concentration: 300 µg/kg/day) were implanted in mice with AAV9-Empty and AAV9-MR, respectively. Aldosterone was also dissolved in saline solution (0.9% NaCl) to ensure proper control. The pumps were explanted after another 14 days, i.e., 4 weeks from the start of the trial. As post-operative care during pump implantation and explantation, animals were given Novalgin via the drinking water (200 mg/kg) for the next three days. After another 14 days, i.e., 6 weeks from the start of the trial or endpoint of the experiment, echocardiographic data were recorded using the Vevo 2100 system (FUJIFILM VisualSonics Inc., Toronto, ON, Canada) before sacrificing the mice. Pressure volume measurements were obtained using a 1F microtip PV catheter (PVR 1045; Millar Instruments, Houston, TX, USA) coupled with a Powerlab/4Sacquisition system (AD instruments, Oxford, UK), maintaining the temperature at 37 °C. For performing various functional assays, hearts were harvested, frozen, and stored at −80 °C. Cardiac functions were analyzed from the echocardiographic data in M-mode and B-mode using VevoLab software (version 5.10.0, FUJIFILM VisualSonics Inc., Toronto, ON, Canada). To randomize and blind the animal study, an internal numbering system was incorporated by a blinded independent researcher. All research conditions and key design features were defined in advance and documented in the approved animal experimental application.

### 2.2. Human Tissue Samples

Heart-failure or healthy control cardiac tissue was collected as previously described [20]. This research was conducted with the endorsement of the institutional ethics committees at the Medical College of Wisconsin in Milwaukee, WI, USA, and the Hannover Medical School in Germany.

### 2.3. Cell Culture

For neonatal rat cardiomyocytes, 1- to 3-day-old rats were sacrificed, and cardiomyocytes were isolated from the explanted hearts following a previously described protocol [21]. For AAV virus production, HEK293T cells were cultured in DMEM (Dulbecco’s Modified Eagle Medium; Gibco, Thermo Fisher Scientific, Waltham, MA, USA) with 4.5 g/L D-Glucose, L-Glutamine, 10% FCS, and 1% penicillin/streptomycin. Aldosterone (Sigma-Aldrich, St. Louis, MO, USA) treatment was conducted for 24 h at a concentration of 1 µM unless stated otherwise. The inhibition of *Ptgds* was obtained using siRNA #mm.si.Ptgds.13.1 (Integrated DNA Technologies, Inc., Coralville, IA, USA) transfection at a concentration of 100 nM using Lipofectamine 2000 (Life Technologies, Thermo Fisher Scientific, Waltham, MA, USA) in OptiMEM medium (Gibco, Thermo Fisher Scientific, Waltham, MA, USA).

### 2.4. RNA and Real-Time PCR

RNA isolation for cell culture samples and tissue samples (from mice) was performed using TriFast (peqGOLD; VWR chemicals, Avantor Inc., Radnor, PA, USA) following the manufacturer’s protocol. The RNA concentration and quality were checked using Multi-Mode Reader Synergy HT (Biotek, Agilent Technologies, Santa Clara, CA, USA), Take 3 session, and Gen5 software (version: 1 November 2005, Biotek). Thereafter, reverse transcription of the isolated RNA (200 ng–1000 ng) was performed using a Biozym cDNA Synthesis Kit (Biozym Scientific GmbH, Hessisch Oldendorf, Germany, Cat. No. 331470) with Oligo dT primers following the manufacturer’s instructions. For quantitative measurement, real-time PCR was performed using SYBR-Green mix (iQ™ SYBR^®^ Green Supermix; Bio-Rad Laboratories, Inc., Hercules, CA, USA; Cat. No. 170-8882) with gene-specific primers (Supplemental Table S2) in an Applied Biosystems ViiA7 instrument (Thermo Fisher Scientific, Waltham, MA, USA).

### 2.5. Western Blotting

Cell lysis buffer (1×) (Cell Signaling Technology, Danvers, MA, USA) along with Pefabloc (Sigma-Aldrich) was used to lyse the cell pellet. Protein quantification was performed using Bradford reagent (Bio-Rad Laboratories). For resolving the proteins, 25 μg of protein per sample was loaded onto SDS polyacrylamide gel along with Precision Plus Protein WesternC standard (Bio-Rad Laboratories). Furthermore, blots were prepared by transferring the proteins from SDS polyacrylamide gel to polyvinylidene fluoride membrane in a Mini PROTEAN Tetra cell (Bio-Rad Laboratories). The following primary antibodies were used to detect the proteins of interest: MR (Abcam; ab2774) and GAPDH (Abcam, Cambridge, UK; ab8245). Secondary antibody conjugated with HRP (anti-mouse IgG, HRP-linked antibody #7076; Cell Signaling Technology) was used for detection of bands.

### 2.6. Adeno-Associated Virus Preparation

MR sequence was obtained from Ensembl Genome browser and was cloned into the pAAV vector using SalI and HindIII (Thermo Fischer Scientific, Waltham, MA, USA). For AAV9 and AAV6 virus production, a previously described protocol was used [22]. To produce AAV9 and AAV6, the pDP9rs or pDP6rs serotype helper plasmids were used, respectively.

### 2.7. RNA-Sequencing and Raw Data Processing

RNA was isolated as described above from *n* = 3 heart tissue samples for AAV9-Empty-Control and AAV9-MR-Aldo mice. The RNA quality was analyzed by using the Bioanalyzer System (Agilent Technologies, Santa Clara, CA, USA) in terms of RNA integrity number (RIN). An Illumina NextSeq 550 sequencer was used to perform the sequencing run at Research Core Unit Genomics (RCUG) of Hannover Medical School.

At first, 500 ng of total RNA per sample was utilized as an input for an rRNA depletion procedure with a ‘NEBNext^®^ rRNA Depletion Kit (Human/Mouse/Rat), 96 rxns’ (E6310X; New England Biolabs, Ipswich, MA, USA), followed by stranded cDNA library generation using a ‘NEBNext^®^ Ultra II Directional RNA Library Prep Kit for Illumina’ (E7760L; New England Biolabs, Ipswich, MA, USA). All steps were performed as recommended in user manual E7760 (Version 1.0_02-2017; NEB), except that all reactions were downscaled to 2/3 of the initial volumes. Furthermore, one additional purification step was introduced at the end of the standard procedure, using 1× ‘Agencourt^®^ AMPure^®^ XP Beads’ (#A63881; Beckman Coulter, Inc., Brea, CA, USA). cDNA libraries were barcoded by a single indexing approach, using ‘NEBNext Multiplex Oligos for Illumina—Set 1’ (Index Primer 2, 4, 5, 6, 7, 12). All generated cDNA libraries were amplified with 9 cycles of final PCR. Fragment length distribution of individual libraries was monitored using a ‘Bioanalyzer High-Sensitivity DNA Assay’ (5067-4626; Agilent Technologies). Quantification of libraries was performed by use of the ‘Qubit^®^ dsDNA HS Assay Kit’ (Q32854; Thermo Fisher Scientific).

Equal molar amounts of six libraries in total were pooled for a common sequencing run. Accordingly, each analyzed library constitutes 16.6% of the overall flowcell capacity. The combined library pool was denatured with NaOH and was finally diluted to 1.5 pM according to the Denature and Dilute Libraries Guide (Document #15048776 v02; Illumina). An amount of 1.3 mL of denatured pool was loaded on an Illumina NextSeq 550 sequencer using a High-Output Flowcell for 75bp paired-end reads (#20024907; Illumina Inc., San Diego, CA, USA). The obtained BCL files were converted to FASTQ files using bcl2fastq Conversion Software version v2.20.0.422 (Illumina).

The FASTQ files were adapter- and quality-trimmed using Trim Galore! (version 0.4.1) with default settings as described in the User Guide except for the setting of the quality cutoff (-q/–quality) which was set to a Phred score of 15. Trim Galore! used Cutadapt (version 1.9.1) as subroutine. Quality control of FASTQ files was performed by FastQC (version 0.11.4) before and after trimming.

After trimming, FASTQ files were mapped against a reference genome with the splice-aware aligner STAR (version 2.5.4a) to generate BAM files. The BAM files were built in a 2-pass mapping (two-pass Mode Basic) and were finally sorted (out SAM type BAM SortedByCoordinate). All other settings were left as default as described in the manual. The genome index files were created by STAR with default settings using *Mus musculus* sequence and annotation data taken from GENCODE.org (GRCm38.p6 release M17).

Quantification was performed with feature counts from the subread package (version 1.6.1) for paired-end reads (-p) with default settings except for the setting of strandedness (-s 2) and the minimum mapping quality score (-Q 10). Annotation data were again taken from GENCODE.org (GRCm38.p6 release M17). Normalization and differential expression analysis was performed with DESeq2 (Galaxy Tool Version 2.11.40.2) with default settings except for “Output normalized counts table”, which was set to “Yes”.

Differentially expressed genes (DEGs) with base mean values > 5 and an adjusted *p*-value < 0.05 were selected for further analysis (Supplemental Table S1). The heat map was generated using the ClustVis online tool [23].

### 2.8. Immunostaining

In order to stain the neonatal rat cardiomyocytes for cell size measurement, 4% paraformaldehyde was used to fix the cells for 10 min followed by permeabilization with 0.1% Triton-X-100 for another 10 min. Blocking was performed in 5% donkey serum for 30 min prior to overnight incubation with primary antibody, namely monoclonal Anti-α-Actinin (Sarcomeric) (A7811; Sigma-Aldrich) at a dilution of 1:500 in 5% donkey serum. Then, cells were incubated with the secondary antibody Anti-Mouse-Alexa 594 (Invitrogen, Waltham, MA, USA) and DAPI for 1 h. In between each step, the cells were washed with DPBS (Gibco) 3 times. Finally, fluorescence microscopy (Nikon Eclipse Ti microscope, Minato (Tokio), Japan) was used to acquire images which were further analyzed with NIS Elements (Nikon) BR imaging software (version 3.22.00).

### 2.9. Immunohistochemistry

For heart tissue from in vivo mice experiments, 5 µm paraffin-embedded heart sections were prepared with a Leica microtome. An additional step of dewaxing and antigen retrieval was performed in freshly prepared citrate buffer (pH 6.0) at a temperature of 96–98 °C for 20 min in case of wheat germ agglutinin (WGA, Invitrogen, Thermo Fisher Scientific, Waltham, MA, USA) staining for cardiomyocyte cell size measurement. Sections were then brought to room temperature and then washed with PBS. After fixation and permeabilization, the sections were stained with Alexa Fluor 488-labeled WGA and DAPI. A Nikon Eclipse Ti microscope was used to obtain images at 20× magnification, which were further analyzed using NIS Elements (version 3.22.00, Nikon, Minato (Tokio), Japan) BR imaging software.

To analyze the fibrosis in heart tissues, Picro-Sirius red staining was performed on paraffin-embedded heart sections. The images were acquired using a Nikon Eclipse Ci microscope with a 2× magnification. Sirius red dye stained the collagen content on the heart section which was then quantified as a percentage of total area of each heart section by using Adobe Photoshop CC2018.

### 2.10. Cell Size Measurement

After immunostaining of NRCMs, images were acquired spanning different areas of respective wells (10–11 images per well). An area of approximately 200 cardiomyocytes was measured using NIS Elements software, and the average value was calculated. The area was normalized to control the samples.

Similarly, for paraffin-embedded heart sections, the images were obtained from 5–6 different regions of the heart. An area of more than 250 cardiomyocytes was measured and averaged per animal. The area was normalized to control the samples.

### 2.11. Statistics

In this study, the experiments were performed 3 or more times as independent biological replicates with three technical replicates in each experiment. We analyzed the data using Prism 7 (Graph Pad) software (version 7.05) and represented it as mean ± SEM throughout the study. An unpaired two-tailed Student’s *t*-test was used for calculating the significance amongst two groups while a one-way ANOVA with Tukey’s post hoc test was performed for more than two groups. We considered a *p*-value < 0.05 as statistically significant in the present study.

## 3. Results

### 3.1. Aldo–MR Pathway Activation Showed Pathological Phenotype in Mice

To understand the impact of an activated Aldo–MR pathway on cardiac physiology, an Adeno-associated virus-9 (AAV9)-based MR overexpression mouse model was generated along with Aldo treatment using osmotic pumps (Figure 1A). The AAV9 serotype of the AAVs possesses a natural tropism for cardiomyocytes in vivo which makes it a suitable serotype under the given experimental conditions. We observed a significant overexpression of MR in mice treated with AAV9-MR as compared to the AAV9-Empty group as analyzed by RT-PCR (Figure 1B). The overexpression was further confirmed at the protein level by immunohistochemistry staining of the cryo-sections from mouse hearts with AAV9-MR-Aldo or AAV9-Empty-Control treatment using Anti-MR antibodies (Figure 1C). Activation of the Aldo–MR pathway was analyzed by the mRNA expression levels of *Lcn2* (NGAL) and *Lgals3* (Gal3) which are well-established downstream effector molecules of an activated Aldo–MR pathway. Confirming the pathway activation, an increase in both the effector molecules was observed (Figure 1B). Echocardiographic analysis of the two experimental groups showed that the Aldo–MR pathway activation caused a decline in cardiac function as observed by a decrease in left ventricular ejection fraction and an increased end-systolic volume (Figure 1D,E; no significant difference in body weight development was observed between the two groups as depicted in Figure S3). Consistently, we detected thickening of the left ventricular anterior wall, along with a significant increase in left ventricular mass upon AAV9-MR-Aldo treatment (Figure S4; Representative echocardiographic M-mode images are shown in Figure S5). However, no signs of diastolic dysfunction were observed at this stage as the IVRT and E/e′ ratio did not differ between the groups (Figure S6).

Furthermore, the mRNA expression levels of myosin heavy chain (MHC) proteins alpha (α) and beta (β) were analyzed by RT-PCR, as a shift in the ratio of these MHC proteins in favor of β-MHC indicates cardiac remodeling. Interestingly, a higher ratio of β-MHC/α-MHC in Aldo-MR overactivation animals as compared to the control animals was observed, indicating the onset of cardiac remodeling (Figure 1F). Since cardiac hypertrophy and fibrosis are major factors contributing towards maladaptive cardiac functioning, WGA and Picro-Sirius red stainings were performed in paraffin sections of the mouse hearts to measure cell size and fibrosis, respectively, in the two animal groups. In line with our previous results, a significant increase in both cell size and fibrosis was observed in animals with Aldo-MR overactivation compared to those in the control group (Figure 1G–J). Thus, our findings suggest that Aldo–MR pathway activation can trigger a diseased cardiac phenotype.

### 3.2. Novel Players Involved in the Aldo–MR-Pathway-Mediated Cardiac Pathology

**Figure 2:**
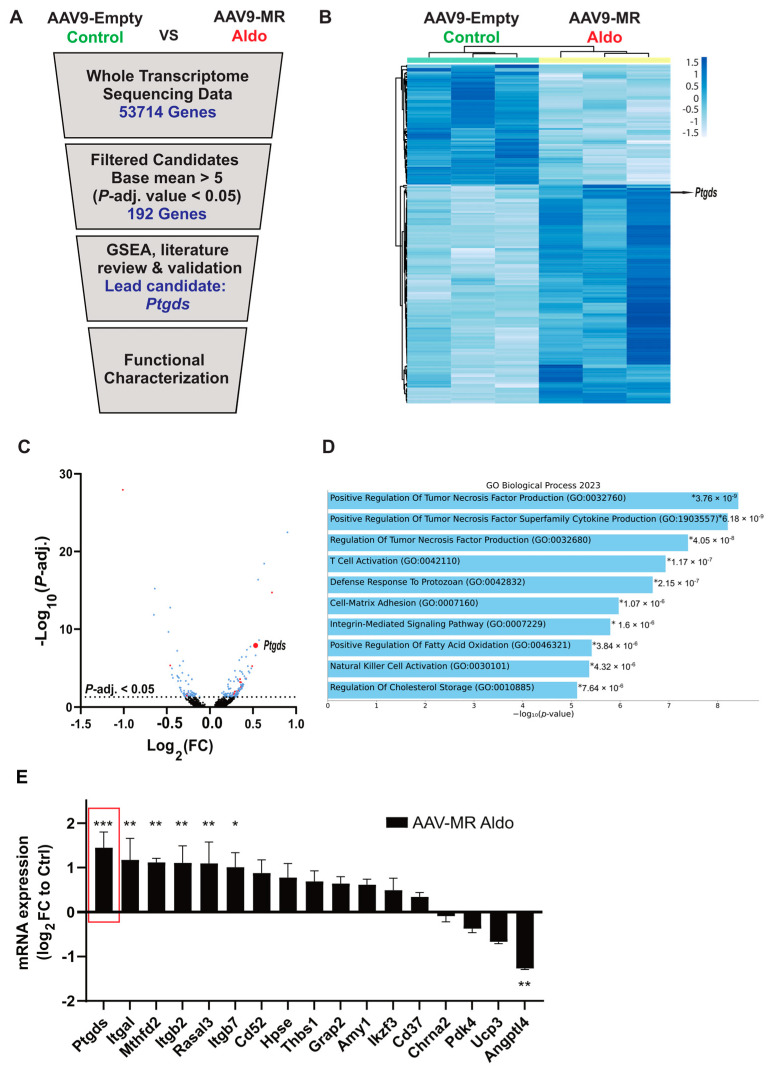
Identification of novel players involved in the Aldo–MR pathway. (**A**) Pipeline used to filter candidates involved in the Aldo–MR pathway using global RNA sequencing of the AAV9-mediated MR-overexpression mice with aldosterone treatment compared to control mice. (**B**) Heat map of 192 significantly differentially regulated genes obtained from the global RNA sequencing with *Ptgds* highlighted in the heat map. Rows are centered and unit variance scaling is applied. Correlation distance and average linkage are used for clustering of both rows and columns. (**C**) Volcano plot showing the significantly up- and down-regulated genes in blue (adjusted *p*-value < 0.05) with the shortlisted candidates highlighted in red. Lead candidate *Ptgds* is labelled and indicated by a larger red point. (**D**) Enriched GO biological processes analyzed using Enrichr. Ranked according to *p*-value. (**E**) mRNA expression of various shortlisted genes in heart tissues from the complete sample set of mice treated with AAV9-Empty-Control and AAV9-MR-Aldosterone as measured by RT-PCR. Gene expression was calculated relative to the housekeeper gene *Gapdh* and normalized to the AAV9-Empty-Control group (*n* = 5–6 animals per group). All data are represented as mean ± SEM. * *p* ≤ 0.05, ** *p* ≤ 0.01, *** *p* ≤ 0.001.

Since a diseased cardiac phenotype was observed in mice with Aldo–MR pathway activation, we aimed to identify novel players driving the pathological signaling mechanism of the Aldo–MR pathway. Therefore, we performed a global RNA sequencing in heart tissue samples from mice transduced either with AAV9-MR or AAV9-Empty, which were further treated with aldosterone or saline (control), respectively (three mice per group). Differentially expressed genes (DEGs) were filtered out by setting a base mean threshold of five, and genes which were significantly (adjusted *p*-value of <0.05) up- or down-regulated in the Aldo–MR-activated group compared to those in the control group were selected (Figure 2A). In total, 125 genes were up-regulated while 67 genes were down-regulated in the MR-overexpression mouse model with Aldo treatment as compared to those of the control animals (Supplementary Table S1). The heat map generated using ClustVis [23] shows the DEGs among the two groups indicating that Aldo–MR pathway activation significantly changes the transcriptome of cardiac tissue from mice (Figure 2B). A volcano plot is shown in Figure 2C, with significantly DEGs indicated above the horizontal line (−log_10_ of an adjusted *p*-value of 0.05). Additionally, gene set enrichment analysis (GSEA) was performed with Enrichr [24,25,26] using the 192 significantly DEGs as the input. Ranked by *p*-value, the top ten enriched biological processes are mostly related to tumor necrosis factor production, inflammatory processes, as well as lipid storage and oxidation (Figure 2D). To shortlist top candidate genes from all DEGs, we performed a literature review to identify possible mediators of the biological processes indicated by GSEA but also of the cardiac pathology observed in our animal model. This approach yielded a list of 17 candidate genes (*Angpl4*, *Amy1*, *Ptgds*, *Itgb2*, *Ucp3*, *Cd37*, *ItgaI*, *Ikzf3*, *RasaI3*, *Cd52*, *Thbs1*, *Mthfd2*, *Itgb7*, *Grap2*, *Pdh4*, *Hpse*, *Chrna2*) that are highlighted in red in the volcano plot (Figure 2C).

To confirm the global sequencing results, these 17 top candidates were validated in independent heart tissue samples by RT-PCR from AAV9-MR-Aldo and AAV9-Empty-Control mice. The expression levels of seven genes were validated to be significantly up- or down-regulated in the complete sample set of the MR-overexpression animal study. We observed a significant increase in the mRNA expression of *Ptgds*, *ItgaI*, *Mthfd2*, *Itgb2*, *RasaI3*, and *Itgb7*, whereas *Angpl4* expression displayed a significant decrease in mice with Aldo–MR-overexpression as compared to control mice (Figure 2E). These genes were selected for further investigations in our in vitro system.

### 3.3. Ptgds Participates in Aldo–MR-Mediated Cardiac Hypertrophy

**Figure 3:**
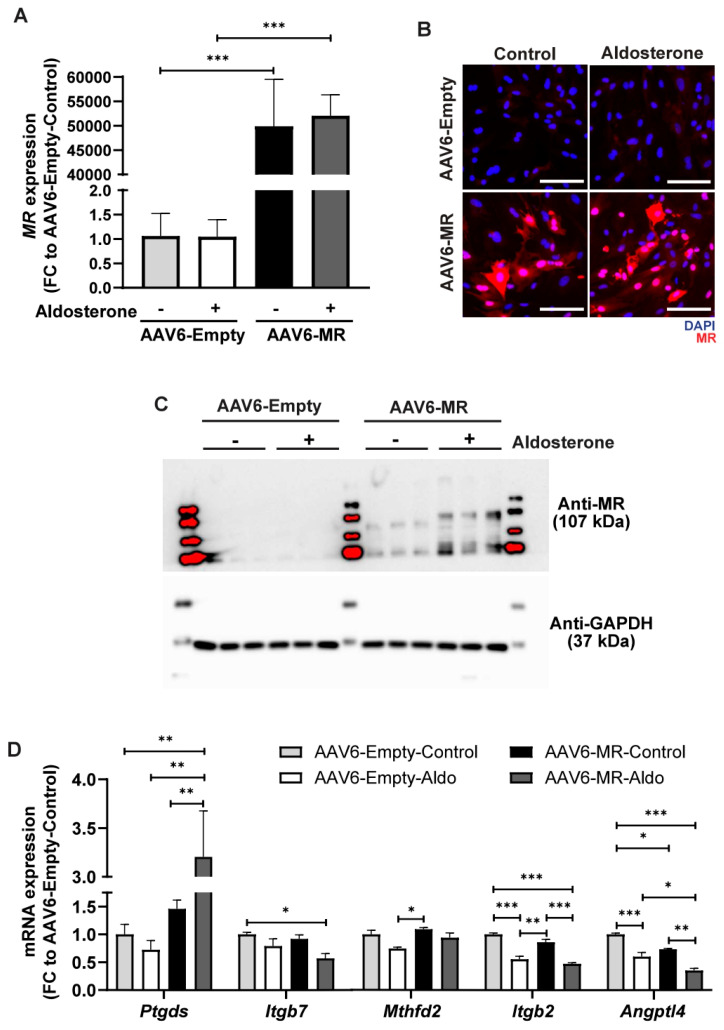
Target validation in NRCMs with AAV6-mediated MR-overexpression and Aldo treatment. (**A**) mRNA expression analysis of MR in NRCMs transduced with AAV6-MR or AAV6-Empty in the presence of Aldo (1 µM) or ethanol control. Gene expression was calculated relative to housekeeper gene *Gapdh* and normalized to the AAV9-Empty control without Aldo treatment. (**B**,**C**) Fluorescent images (**B**) of AAV6-mediated MR-overexpression in NRCMs (MR (red) and DAPI (blue)) and Western blots incubated with Anti-MR and Anti-GAPDH antibodies (**C**) of lysates from NRCMs after transduction with AAV6-Empty or AAV6-MR in the presence of Aldo (1 µM) or ethanol control. (**D**) mRNA expression of the detectable top candidate genes as measured in NRCMs transduced with AAV6-MR or AAV6-Empty in the presence of Aldo (1 µM) or ethanol via RT-PCR. Gene expression was calculated relative to housekeeper *Gapdh* and normalized to AAV6-Empty-Control. Scale bar indicates 100 µm. All data are represented as mean ± SEM. *n* = 3, * *p* ≤ 0.05, ** *p* ≤ 0.01, *** *p* ≤ 0.001.

To better understand the mechanism of action in the Aldo–MR pathway, we incorporated an in vitro system that mimics our Aldo–MR over-activation mouse model. In contrast to cell lines, normal cell morphology and function observed in vivo are preserved in primary cells. Therefore, we used neonatal rat cardiomyocytes (NRCMs) which provide an excellent in vitro system for the characterization of functional and molecular mechanisms in the heart. An AAV6-mediated MR-overexpression was performed in NRCMs to investigate mechanistic involvement of the identified top candidates. The schematic plasmid map of pAAV-MR used to achieve the overexpression of MR both in vitro and in vivo is shown in Supplementary Figure S1. NRCMs were transduced with AAV6-MR or AAV6-Empty with MOIs = 5 × 10^4^ for 48 h and were further treated with 1 µM Aldo or ethanol as control for 24 h, and the mRNA expression of various candidate genes was measured via RT-PCR. Robust MR overexpression in NRCMs was validated by RT-PCR, immunofluorescence, and Western blotting (Figure 3A−C).

Similar to our previous results in vivo, the expression of *Ptgds* was highly increased in NRCMs with AAV6-mediated MR-overexpression which increased further by Aldo treatment (Figure 3D). Interestingly, 1 µM Aldo treatment alone did not increase the expression of *Ptgds* in the AAV6-Empty control group. Among the other genes that could be detected in the in vitro model, only *Angptl4* showed a similar trend as observed in the animal study where the transduction of AAV6-MR led to a significant reduction in expression, while the treatment with Aldo further reduced the *Angptl4* expression compared to that of the control (Figure 3D).

In line with our findings, *Ptgds* was previously identified as a direct downstream target of MR in adipocytes [27]; however, the specific role of *Ptgds* in the context of the Aldo–MR pathway in cardiomyocytes is still unknown. *Angptl4* also showed promising potential as it has an inverse correlation with MR overexpression which makes it an ideal candidate for *Angptl4*-overexpression-based therapies. However, a literature survey of *Angptl4* was contradictory, showing a positive association with cardiac diseases rather than having cardio-protective effects. For example, increased levels of *Angptl4* were observed in cardiometabolic disorders including atherosclerosis and type 2 diabetes while a loss of function genetic variant of *AngptI4* reduced the risk for coronary artery disease. Therefore, we selected *Ptgds* for further investigation in the in vitro system.

### 3.4. Ptgds Is Involved in Cardiac Pathologies

Since *Ptgds* expression levels were up-regulated upon Aldo–MR pathway activation, we hypothesized that its inhibition might reverse the cardiac disease phenotype. Hence, we performed an siRNA-mediated *Ptgds* inhibition in NRCMs. Three different siRNAs against *Ptgds* (siPtgds13.1, 13.2, 13.3) were tested for their inhibition efficacy of *Ptgds* as compared to a siRNA-negative control (siCtrl) by RT-PCR (Supplementary Figure S2). SiRNA13.1 was selected for future experiments owing to its high inhibition efficiency for *Ptgds*.

**Figure 4:**
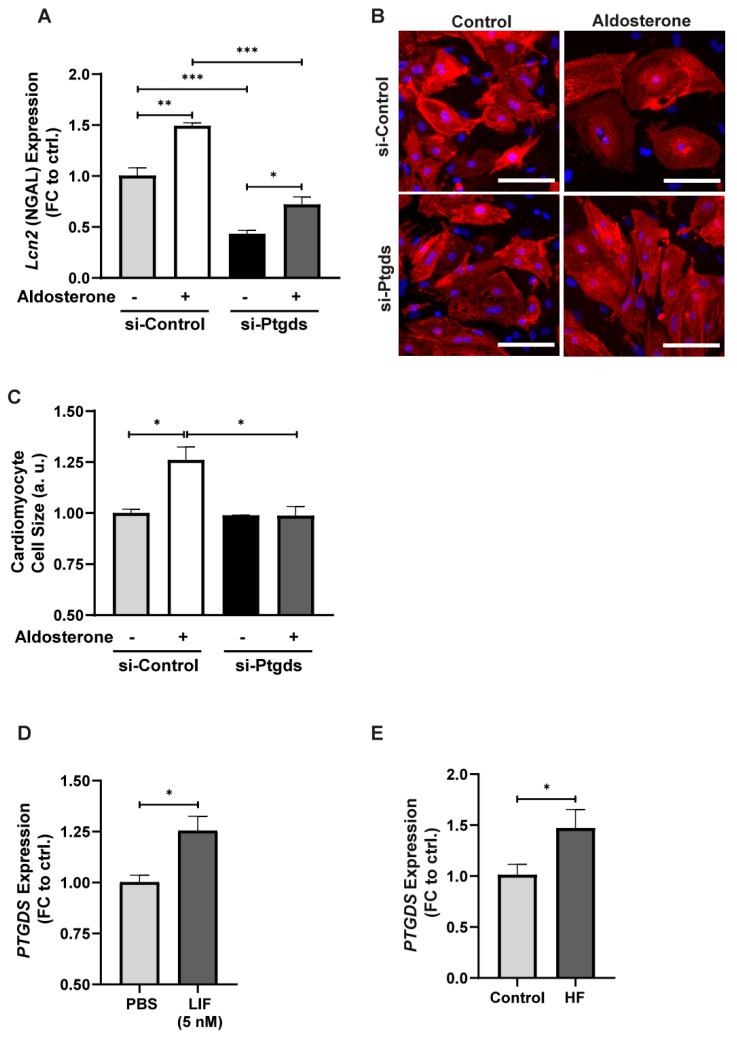
*Ptgds* involved in cardiac pathologies. (**A**) *Lcn2* (NGAL) mRNA expression after transfection of NRCMs with siPtgds or siCtrl at a concentration of 100 nM and ethanol/Aldo treatment (0.1 µM) as measured by RT-PCR. (**B**,**C**) Fluorescent images (**B**) obtained after α-actinin (red) and DAPI (blue) immunostaining of NRCMs and cardiomyocyte cell size measurement (**C**) after siPtgds or siCtrl transfection (100 nM) and ethanol/Aldo treatment (0.1 µM) (*n* = 2). (**D**,**E**) mRNA expression of *PTGDS* after the treatment with 5 nM LIF or control (PBS) in hiPSC-CMs (*n* = 3) (**D**) and in heart tissue samples from patients with end-stage heart failure and controls (*n* = 22/12) (**E**) as measured by RT-PCR. All data are represented as mean ± SEM. * *p* ≤ 0.05, ** *p* ≤ 0.01, *** *p* ≤ 0.001. Scale bar indicates 100 μm. a.u., arbitrary units.

The expression of *Lcn2* (NGAL) was measured as a marker for MR pathway activation. After si-Ptgds transfection, the expression of NGAL was significantly decreased in comparison to the si-Control treated cells. Importantly, si-*Ptgds* treatment rescued the levels of NGAL expression also after Aldo treatment (Figure 4A), showing the effect of *Ptgds* inhibition on the Aldo–MR pathway.

Since Aldo–MR activation leads to a hypertrophic phenotype, as observed in our animal study, we analyzed if *Ptgds* inhibition could reverse this effect. Interestingly, there was no significant change in cell size under control conditions without Aldo stimulation and *Ptgds* inhibition. However, under Aldo treatment, the hypertrophic effect was significantly reversed by the inhibition of *Ptgds* indicating that *Ptgds* specifically alters Aldo induced hypertrophy and did not have apparent effects under physiological conditions (Figure 4B,C). These results suggest the involvement of *Ptgds* in the hypertrophic effects of the Aldo–MR pathway.

For further validation, the expression of *PTGDS* was measured in human-induced pluripotent stem cell (iPSC)-derived cardiomyocytes (hiPSC-CM) after treatment with 5 nM LIF or PBS as control via RT-PCR. LIF (leukemia inhibitory factor) is a pleiotropic cytokine that is known to induce cardiomyocyte hypertrophy [28]. *PTGDS* expression was significantly increased in hiPSCs-CMs after LIF treatment as compared to that of the control (Figure 4D), corroborating the notion that *PTGDS* is involved in the development of cardiac hypertrophy even in human-based models, suggesting translational importance of *PTGDS* as a therapeutic target in Aldo–MR-derived cardiac remodeling.

Furthermore, the expression of *PTGDS* was measured in heart tissue samples from patients with end-stage heart failure compared to control patients. Consolidating our previous results, we observed a significant increase in the expression of *PTGDS* in patients with end stage heart failure as compared to controls. Thus, *PTGDS* shows high potential to be developed as a therapeutic target against Aldo–MR-mediated cardiac pathologies.

## 4. Discussion

An over-activated Aldo–MR pathway has been associated with cardiovascular conditions such as hypertension, myocardial infarction, and subsequent heart failure [2]. Therefore, we aimed to identify novel molecular targets and to characterize their potential for interference with pathological Aldo–MR signaling.

Using an AAV9-mediated MR overexpression system activated with Aldo-infused pumps, a diseased cardiac phenotype consisting of cellular hypertrophy, fibrosis, and deteriorated cardiac functions was observed showing that an abnormally activated Aldo–MR pathway has severe detrimental impacts on cardiac health. In line with our observations, a study using cardiac-specific hMR overexpression identified a critical coronary endothelial dysfunction [29] while another study associated it with ventricular arrhythmias which was prevented by an MR antagonist [30]. Although the AAV9 serotype is considered the gold standard for in vivo cardiac transgene delivery in mice [31], a high expression of transgenes is observed in the liver, lungs, and kidneys [32]. Therefore, caution is warranted, as a contribution of extra-cardiac MR to the disease phenotype cannot be ruled out. Thus, cardiac-specific promoters such as α-MHC or cTnT in place of the CMV promoter would reduce the extra-cardiac MR overexpression aiding the investigation of cardiac-specific effects. Moreover, despite not being the primary focus of the present study, follow-up investigations might also want to evaluate the effects of MR overexpression with Aldo stimulation on the blood pressure of mice.

Global RNA sequencing of heart tissue samples from our animal model identified *Ptgds* as a key player in the Aldo–MR pathway, which was found to be up-regulated with over-activation of the Aldo–MR pathway in cardiomyocytes. Urbanet et al. previously identified PTGDS as a downstream target of MR in adipocytes [27], which is consistent with our findings in cardiac settings. We further confirmed its role in Aldo-mediated hypertrophy where *Ptgds* inhibition reduced the hypertrophic effect of Aldo on NRCMs. The inhibition of *Ptgds* also decreased the levels of *Lcn2* (NGAL), suggesting its involvement in NGAL-mediated cardiac pathologies. Even though the association between *Ptgds*- and Aldo–MR-related cardiac diseases has not been explored much, its expression is elevated in myocardial infarction [33] and it is advocated as a biomarker for cardiovascular risk prediction due to its association with heart failure and acute coronary syndrome [34]. Interestingly, we also observed an increase in *PTGDS* in hiPSC-CMs treated with LIF, a pro-hypertrophic compound [28], as well as in patients with end-stage heart failure. These results consolidate the importance of *Ptgds* as a pivotal factor in cardiovascular pathologies. Additionally, GSEA revealed that the overactivation of the Aldo–MR pathway in the present mouse experiments led to the enrichment of genes associated with biological processes related to the activation of the immune system and the production of tumor necrosis factor (TNF) and TNF superfamily cytokines. Interestingly, a potential link between these biological processes and the observed general cardiac pathologies, such as cardiomyocyte hypertrophy and cardiac fibrosis, might be found in our lead candidate gene, *Ptgds*. Specifically, the gene *Ptgds* encodes for prostaglandin D synthase, responsible for synthesizing one of many bioactive prostanoid lipids termed prostaglandin D2 (PGD2) [35]. The connection between prostanoids and inflammation [36,37], including TNF synthesis and signaling [38,39,40] is well established in the literature. Depending on the cell type and receptor PGD2 in particular acts upon, it exhibits both pro- and anti-inflammatory effects. In the heart specifically, both beneficial and detrimental effects are associated with PGD2 [41]. Adding to the complexity, Fitzpatrick et al. demonstrated the rapid conversion of PGD2 into active metabolites [42]. One such metabolite is Prostaglandin F 2 alpha (PGF2α) that can be produced by prostaglandin F synthase from PGD2 [43]. Notably, PGF2α is known to induce cardiomyocyte hypertrophy in vitro [44], and stimulation of the PGF2α receptor (FP) in vivo leads to hypertrophic hearts. Moreover, in cardiac fibroblasts, PGF2α is a contributor to myocardial fibrosis by stimulating collagen deposition [45]. Future studies should focus on evaluating whether direct targeting of *Ptgds* or, perhaps, a more specific pharmacological intervention, such as FP receptor inhibition, could prove to be a valid therapeutic strategy in the context of Aldo–MR-pathway-related cardiac pathologies. Encouragingly, a study of FP receptor gene silencing suggested that it can ameliorate diabetic cardiomyopathy and myocardial fibrosis [46], making it a promising avenue for further studies.

This study has some limitations. First, the experimental design did not include additional control groups such as AAV9-Empty-Aldo and AAV9-MR-Control, which restricts certain comparisons. Second, blood pressure was not directly assessed, limiting our ability to link structural and functional changes to hemodynamic alterations. Instead, we invasively determined the maximum left ventricular pressure (P_max_; Figure S6). However, this is a contractility-related parameter and not a proxy for systolic pressure. Although not the primary focus of the present study, future investigations should also evaluate the impact of MR overexpression combined with Aldo stimulation on blood pressure to better understand its contribution to cardiac remodeling. Finally, mechanistic insights into the regulation of PTGDS expression were beyond the scope of present study, which should be addressed in future studies.

Given the demonstrated involvement of *Ptgds* in regulating glucose transport, insulin resistance, and glucose intolerance under diabetic conditions [47], it is pertinent to further explore these metabolic effects of *Ptgds* with regards to the Aldo–MR pathway and cardiac remodeling. Moreover, an AAV-mediated *Ptgds* overexpression study in combination with a detailed investigation of its effects on prostanoid levels would be useful in broadening our understanding of its effects on cardiac health and identifying novel effector molecules of this pathway.

## 5. Conclusions

Our findings demonstrate that sustained activation of the MR–Aldo pathway drives adverse cardiac remodeling, characterized by fibrosis, hypertrophy, and impaired function, validating AAV9-MR-Aldo as a relevant mouse model. Transcriptomic profiling identified *Ptgds* as a candidate mediator of this process, and functional experiments showed that silencing *Ptgds* reversed hypertrophy in vitro. Importantly, elevated *PTGDS* expression was also observed in human iPSC-derived cardiomyocytes and in patients with end-stage heart failure, underscoring its translational relevance. Future studies should clarify the mechanisms regulating PTGDS expression and its metabolic consequences, which may open new therapeutic avenues in heart failure.

## Data Availability

RNA-sequencing data will be made publicly available via the GEO database under the accession number GSE308485, or following this link: https://www.ncbi.nlm.nih.gov/geo/query/acc.cgi?acc=GSE308485 (accessed on 14 September 2025).

## Supplementary Materials

The following supporting information can be downloaded at: https://www.mdpi.com/article/10.3390/cells14191485/s1, Figure S1: Vector map of pAAV-CMV-MR; Figure S2: Analyzing *Ptgds* expression after its siRNA-mediated inhibition. Figure S3: Change in body weight over the course of the experiment; Figure S4: Left ventricular parameters point towards cardiac remodeling; Figure S5: Representative echocardiographic M-mode images; Figure S6: Parameters of hemodynamic and diastolic function; Table S1: List of differentially expressed genes; Table S2: Gene-specific primers for qPCR.

## Abbreviations

The following abbreviations are used in this manuscript:

AAV	Adeno-associated virus
CVD	Cardiovascular disease
DEGs	Differentially expressed genes
FP	Prostaglandin F2 alpha receptor
GSEA	Gene set enrichment analysis
hiPSC-CMs	Human-induced pluripotent stem cell-derived cardiomyocytes
LIF	Leukemia inhibitory factor
MI	Myocardial infarction
MR	Mineralocorticoid receptor
PGD2	Prostaglandin D2
PGF2α	Prostaglandin F2 alpha
Ptgds	Prostaglandin D2 synthase
siRNA	Small interfering ribonucleic acid
TNF	Tumor necrosis factor
