# Supplementary material for: Prostaglandin D2 Synthase: A Novel Player in the Pathological Signaling Mechanism of the Aldosterone–Mineralocorticoid Receptor Pathway in the Heart
Source: Cells. 2025 Sep 23;14(19):1485. doi: 10.3390/cells14191485 (PMC12523245; doi:10.3390/cells14191485)
Supplement: Supplementary file 1 [file cells-14-01485-s001.zip › supplemental_figures_revision_cells.pdf]

Suporting information – Supplemental material

Prostaglandin D_2 Synthase: A novel Player in the Pathological Signaling Mechanism of the Aldosterone–Mineralocorticoid Receptor Pathway in the Heart

Ankita Garg [1,†], Malte Juchem [1, 2,†], Sinje Biss [1], Carla Nunes Borisch [1], Julia Leonardy [1], Christian Bär [1, 2, 3,‡], Shashi Kumar Gupta [1,‡, §], and Thomas Thum [1,‡,*]

1 Institute of Molecular and Translational Therapeutic Strategies (IMTTS), Hannover Medical School, 30625 Hannover, Germany

2 Fraunhofer Institute for Toxicology and Experimental Medicine (ITEM), 30625 Hannover, Germany

3 Fraunhofer Cluster of Excellence Immune-Mediated Diseases (CIMD), 30625 Hannover, Germany

* Correspondence: thum.thomas@mh-hannover.de

† These authors contributed equally to this work

‡ These authors jointly directed the work

§ Current address: Council of Scientific and Industrial Research - Central Drug Research Institute, Lucknow 226031, India

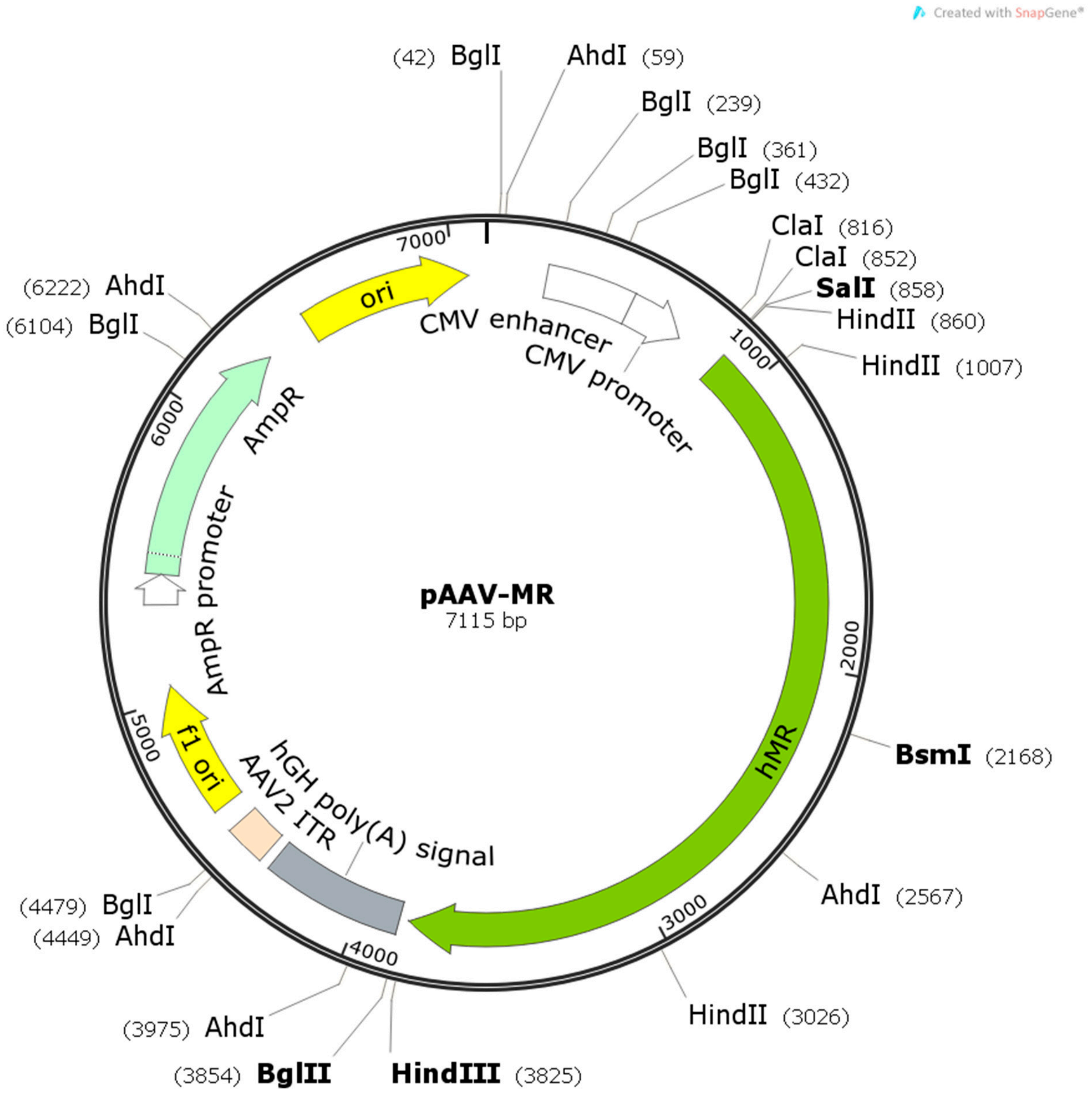

Figure S1 – Vector map of pAAV-CMV-MR

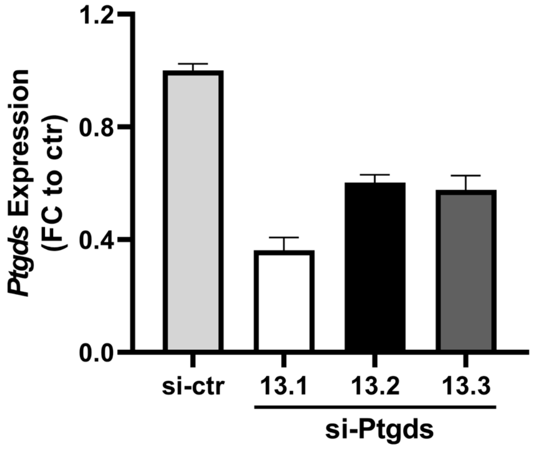

Figure S2 – Analyzing Ptgds expression after its siRNA-mediated inhibition. Ptgds mRNA expression ana-lysed by RT-PCR after transfection of NRCMs with three different siPtgds (13.1, 13.2, 13.3) at a concentration of 100 nM as compared to siCtrl. All data are represented as mean ± SEM, N=1, three technical replicates

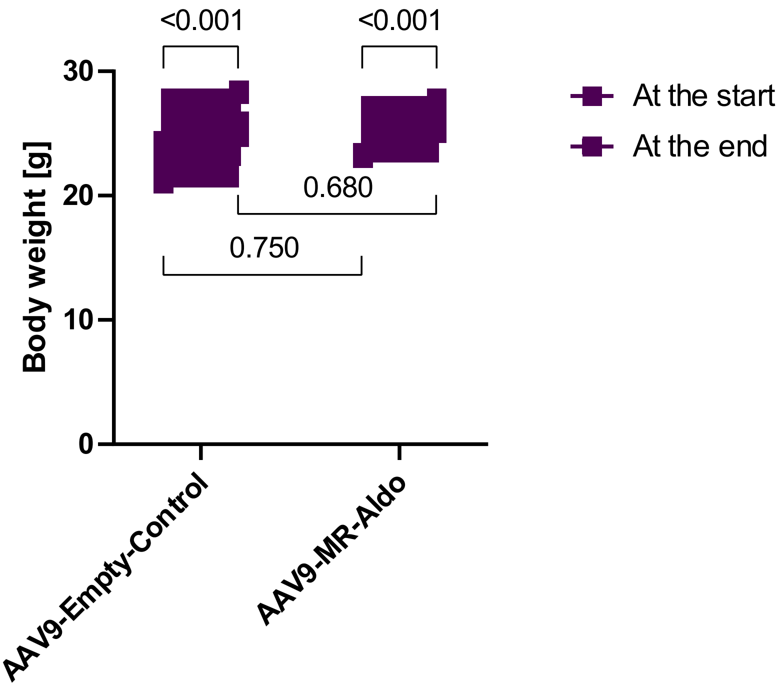

Figure S3 – Change in body weight over the course of the experiment. Body weight increased in both groups from baseline to endpoint. n=5 for control group and n=6 for AAV9-MR-Aldo group. Two-way ANOVA, followed by Sidak's multiple comparisons test.

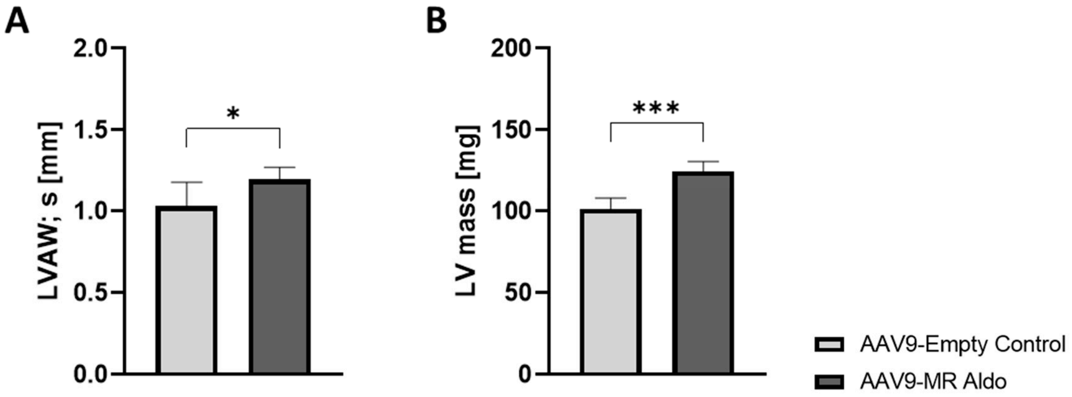

Figure S4 – Left ventricular parameters point towards cardiac remodeling. (A) Left ventricular anterior wall thickness (LVAW) increases in the AAV9-MR overexpression Aldo group compared to control animals. (B) AAV9-MR Aldo animals present a significant increase in left ventricular (LV) mass. n=5 for control group and n=6 for AAV9-MR-Aldo group. Data are presented as mean ± SD. Statistical significance was determined using unpaired t-test.

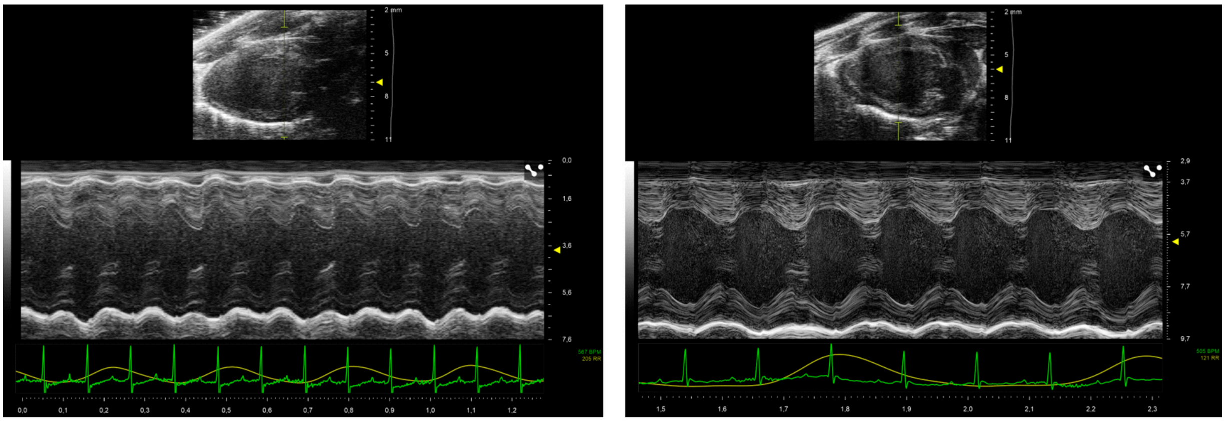

AAV9 Empty-Control | AAV9 MR-Aldo

Figure S5 – Representative echocardiographic M-mode images.

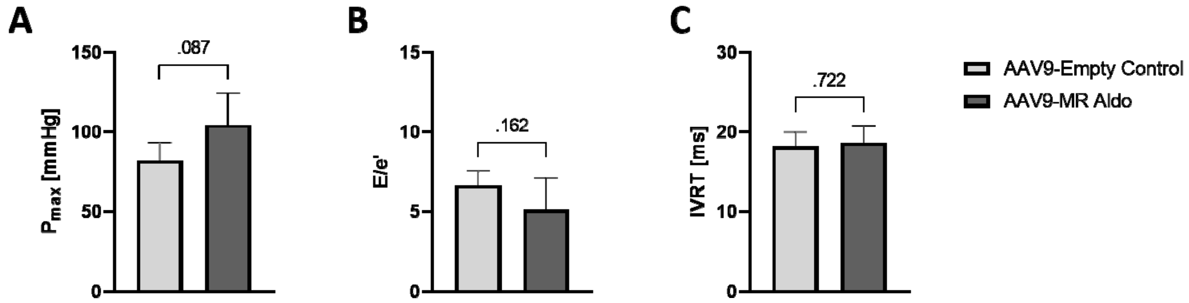

Figure S6 – Parameters of hemodynamic and diastolic function. (A) Maximum left ventricular pressure (Pmax) shows an increasing trend in the AAV9-MR Aldo group compared to AAV9-Empty control, n=4 for AAV9-Empty control and n=5 for AAV9-MR Aldo. (B) No statistically. significant difference in E/e′ between the two groups. n=5 for AAV9-Empty control and n=5 for AAV9-MR Aldo. (C) Isovolumetric relaxation time (IVRT) shows no significant difference between the groups. n=5 for AAV9-Empty control and n=6 for AAV9-MR Aldo. Data are presented as mean ± SD. Statistical significance was determined using unpaired t-test.
