# Supplementary material for: Prostaglandin D2 Synthase: A Novel Player in the Pathological Signaling Mechanism of the Aldosterone–Mineralocorticoid Receptor Pathway in the Heart
Source: Cells. 2025 Sep 23;14(19):1485. doi: 10.3390/cells14191485 (PMC12523245; doi:10.3390/cells14191485)
Supplement: Supplementary file 1 [file cells-14-01485-s001.zip › SupplementaryTables.pdf]

Supplemental Table S1: List of differentially expressed genes

Gene name	Base mean	log_2(Fold change)	Standard error	Wald-Stats	P-value	P-adjusted
Angptl4	193	-1.01	0.08	-11.96	5.96E-33	1.14E-28
Lcn2	151	0.90	0.08	10.80	3.5206E-27	3.3548E-23
Nppa	5304	0.63	0.06	9.87	5.5707E-23	3.5389E-19
Acta1	6847	0.56	0.06	9.35	8.7011E-21	4.1456E-17
Lgals4	1806	-0.64	0.07	-9.04	1.5454E-19	5.8906E-16
Amy1	390	0.72	0.08	8.89	5.8667E-19	1.8635E-15
Cpt1a	3185	-0.46	0.05	-8.36	6.17E-17	1.6803E-13
Scd4	393	-0.65	0.08	-8.09	6.1343E-16	1.4614E-12
Slc25a22	1320	-0.48	0.07	-7.44	1.04E-13	2.21E-10
Dock2	435	0.57	0.08	7.09	1.35E-12	2.57E-09
Ptgds	4796	0.53	0.08	6.85	7.29E-12	1.26E-08
Tnni2	47	0.47	0.07	6.80	1.0788E-11	1.7134E-08
Pfkfb2	2565	0.43	0.06	6.64	3.0705E-11	4.5014E-08
Gpam	8879	-0.38	0.06	-6.59	4.40E-11	5.9874E-08
H2-Q6	647	0.53	0.08	6.38	1.8211E-10	2.3138E-07
Ppp1r3c	3321	0.42	0.07	6.30	2.9893E-10	3.5606E-07
Ankrd1	26207	0.37	0.06	6.28	3.40E-10	3.8061E-07
Ccl5	43	0.34	0.06	6.13	9.0517E-10	9.5837E-07
Slc27a1	7504	-0.31	0.05	-6.07	1.2693E-09	1.2732E-06
Ucp3	1911	-0.46	0.08	-5.85	4.9134E-09	4.682E-06
Aldh9a1	1258	-0.35	0.06	-5.83	5.4051E-09	4.9052E-06
Laptm5	691	0.44	0.08	5.80	6.7708E-09	5.6104E-06
Itgb2	326	0.49	0.08	5.80	6.58E-09	5.61E-06
Ctss	430	0.48	0.08	5.67	1.4432E-08	1.1002E-05
RP24-488I7.2	534	-0.44	0.08	-5.67	1.4246E-08	1.1002E-05
Nkg7	24	0.29	0.05	5.54	3.07E-08	2.25E-05
Il2rb	58	0.35	0.06	5.52	3.451E-08	2.4359E-05
Ptprc	793	0.45	0.08	5.50	3.9056E-08	2.6583E-05
Itgax	57	0.37	0.07	5.45	5.041E-08	3.3128E-05
Nr4a3	379	0.46	0.08	5.41	6.1864E-08	3.93E-05
Slc25a42	3102	-0.32	0.06	-5.40	6.80E-08	4.18E-05
Gimap3	58	0.38	0.07	5.33	9.8201E-08	5.8485E-05
Acot1	266	-0.44	0.08	-5.28	1.3054E-07	7.539E-05
Mylk4	12724	-0.25	0.05	-5.25	1.4912E-07	8.3585E-05
Apex2	1408	-0.38	0.07	-5.21	1.86E-07	0.00010132
H2-Q4	1123	0.40	0.08	5.19	2.1437E-07	0.00011349

Prrg1	825	-0.37	0.07	-5.13	2.851E-07	0.00014611
Cited1	25	0.30	0.06	5.13	2.9134E-07	0.00014611
Abhd1	152	-0.43	0.08	-5.08	3.76E-07	1.84E-04
Slc40a1	901	-0.36	0.07	-5.07	3.9398E-07	0.00018771
Cd37	124	0.42	0.08	5.04	4.6746E-07	0.00021564
Itgal	184	0.41	0.08	5.04	4.7523E-07	0.00021564
Coro1a	312	0.42	0.08	5.01	5.5491E-07	0.00024594
Ikzf3	64	0.35	0.07	4.99	5.96E-07	2.58E-04
Frmd5	4220	0.32	0.06	4.98	6.4775E-07	0.00027069
Lbh	23284	-0.21	0.04	-4.97	6.53E-07	2.71E-04
Gzmb	21	0.22	0.04	4.95	7.32E-07	2.97E-04
Myo5c	772	-0.34	0.07	-4.88	1.0466E-06	0.00041555
Nppb	3185	0.33	0.07	4.88	1.0865E-06	0.00041722
Fam189a2	1730	-0.28	0.06	-4.87	1.0946E-06	0.00041722
Cxcr6	22	0.24	0.05	4.86	1.1726E-06	0.00043817
Pfkfb1	1061	-0.37	0.08	-4.85	1.22E-06	0.00044799
Ms4a4b	38	0.28	0.06	4.84	1.3313E-06	0.00047872
Rasal3	58	0.35	0.07	4.79	1.6458E-06	0.00058086
Cd52	70	0.36	0.07	4.77	1.87E-06	6.50E-04
Cotl1	335	0.38	0.08	4.67	3.0343E-06	0.00103263
Rnf207	4354	-0.25	0.05	-4.65	3.3612E-06	0.00112381
Klrc1	16	0.21	0.05	4.63	3.6952E-06	0.0012142
Gbp4	912	0.36	0.08	4.61	3.9361E-06	0.00125024
Gck	294	0.39	0.08	4.61	3.931E-06	0.00125024
Stat1	804	0.37	0.08	4.57	4.9508E-06	0.00154677
Myo1f	238	0.38	0.08	4.52	6.11E-06	0.00187679
Cybb	580	0.36	0.08	4.50	6.75E-06	2.04E-03
Myo1g	178	0.38	0.08	4.49	7.20E-06	2.14E-03
Nek10	15	0.21	0.05	4.47	7.9031E-06	0.00231719
Ciita	209	0.37	0.08	4.43	9.39E-06	2.71E-03
Thbs1	1245	0.36	0.08	4.43	9.5553E-06	0.00271799
Vwa3a	415	-0.35	0.08	-4.39	1.1221E-05	0.00312259
Fermt3	238	0.37	0.08	4.39	1.13E-05	0.00312259
RP23-458G12.4	539	-0.34	0.08	-4.38	1.2077E-05	0.00328799
Sh3bgrl3	408	0.34	0.08	4.36	1.3101E-05	0.00351652
Clu	9568	0.24	0.06	4.33	1.49E-05	3.93E-03
Psmb8	358	0.37	0.08	4.32	1.58E-05	0.0041173
Rgs1	20	0.22	0.05	4.31	1.6399E-05	0.00422342
Gbp3	543	0.36	0.08	4.28	1.8564E-05	0.00468876

Lcp1	1061	0.33	0.08	4.28	1.87E-05	4.69E-03
Ivns1abp	46243	0.22	0.05	4.27	1.97E-05	4.88E-03
Gbp2	667	0.35	0.08	4.23	2.3665E-05	0.00578211
RP23-458G12.3	665	-0.34	0.08	-4.21	2.5363E-05	0.00611856
Ccr2	165	0.34	0.08	4.17	2.9891E-05	0.00712075
Aqp7	1374	-0.30	0.07	-4.16	3.2212E-05	0.00757894
Mthfd2	75	0.33	0.08	4.14	3.41E-05	7.92E-03
Rac2	120	0.34	0.08	4.14	3.451E-05	0.00792392
Abcd3	4702	-0.22	0.05	-4.12	3.7383E-05	0.00848142
Acot2	2332	-0.25	0.06	-4.12	3.81E-05	8.53E-03
Abra	1020	0.31	0.08	4.11	4.0115E-05	0.00888968
Selplg	125	0.34	0.08	4.09	4.2259E-05	0.00925723
Lgals3bp	1088	0.30	0.07	4.09	4.3979E-05	0.00952444
Itgb7	54	0.29	0.07	4.07	4.68E-05	1.00E-02
RP23-458G12.2	518	-0.33	0.08	-4.06	4.9127E-05	0.010403
Pdcd1	10	0.16	0.04	4.05	5.1111E-05	0.0107041
C1qb	895	0.32	0.08	4.04	5.4237E-05	0.01123537
Bri3bp	1292	-0.25	0.06	-4.00	6.2178E-05	0.01274179
Mhrt	7716	-0.20	0.05	-4.00	6.327E-05	0.01282775
Lrrc10	5297	0.27	0.07	3.99	6.7325E-05	0.01350604
Irs1	3330	-0.21	0.05	-3.98	6.9105E-05	0.01371869
RP23-458G12.1	778	-0.30	0.08	-3.96	7.5554E-05	0.01484437
Gbp2b	11	0.14	0.04	3.94	8.1812E-05	0.01576113
Irf2bpl	2149	0.24	0.06	3.94	8.1874E-05	0.01576113
Adgre1	572	0.31	0.08	3.92	8.67E-05	1.65E-02
Lck	24	0.21	0.05	3.92	8.82E-05	1.66E-02
Hnrnpl	6030	-0.19	0.05	-3.91	9.16E-05	1.68E-02
Grap2	54	0.28	0.07	3.91	9.1864E-05	0.0168341
Ehhadh	472	-0.30	0.08	-3.92	9.03E-05	1.68E-02
Itga4	374	0.33	0.08	3.91	9.3787E-05	0.01686217
Mef2d	7283	-0.18	0.05	-3.91	9.36E-05	1.69E-02
Trim63	5340	0.18	0.05	3.89	9.9089E-05	0.01748878
RP23-42D10.5	143	-0.31	0.08	-3.89	9.9184E-05	0.01748878
Lgals3	83	0.30	0.08	3.89	0.00010003	0.01748878
Spn	80	0.27	0.07	3.88	0.00010331	0.01789909
Gpr75	48	-0.23	0.06	-3.88	0.000105	0.01802761

Mllt3	788	-0.27	0.07	-3.87	0.0001108	0.01885379
Pdk4	9347	-0.26	0.07	-3.86	1.15E-04	1.91E-02
RP23-93K3.8	6220	-0.19	0.05	-3.86	0.00011364	0.01907525
Etv5	728	0.27	0.07	3.86	1.15E-04	1.91E-02
Apob	13	-0.16	0.04	-3.85	1.16E-04	1.91E-02
Klrk1	22	0.20	0.05	3.85	0.00011871	0.01933598
Sla	150	0.31	0.08	3.84	1.22E-04	1.96E-02
Pik3r1	7970	0.23	0.06	3.84	0.0001245	0.01993946
Hk3	77	0.31	0.08	3.83	0.00012749	0.02024807
Cd3g	14	0.16	0.04	3.81	1.38E-04	2.18E-02
Rnf144a	910	-0.26	0.07	-3.81	1.40E-04	2.19E-02
Hpse	57	0.28	0.07	3.80	0.00014358	0.02224601
Btnl9	2529	-0.22	0.06	-3.79	0.00014844	0.02281484
Igha	12	0.16	0.04	3.79	0.00015043	0.02293465
Hsd17b11	752	-0.28	0.07	-3.79	0.00015314	0.02316302
Sh2d2a	20	0.18	0.05	3.78	0.00015716	0.02331375
Col1a2	7434	0.19	0.05	3.78	0.00015662	0.02331375
Lilr4b	252	0.32	0.08	3.78	0.00015781	0.02331375
Adcy7	950	0.26	0.07	3.77	0.0001663	0.02437976
Tbc1d4	5311	-0.20	0.05	-3.76	0.00017053	0.02480883
Pf4	326	0.32	0.08	3.76	0.00017324	0.02483313
RP23-476E21.3	75	-0.30	0.08	-3.76	0.0001733	0.02483313
Ech1	22453	-0.17	0.05	-3.75	0.00017604	0.02503649
Irf8	304	0.31	0.08	3.74	0.00018279	0.02580474
Ctla2a	408	0.32	0.08	3.74	0.00018564	0.02601472
B4galnt1	82	0.29	0.08	3.73	1.95E-04	2.70E-02
Cd274	463	0.31	0.08	3.73	1.95E-04	2.70E-02
Trbc2	25	0.17	0.05	3.71	0.00020617	0.02826772
Scn4b	1364	-0.27	0.07	-3.70	0.00021343	0.02905396
Mn1	1422	0.23	0.06	3.68	0.00023546	0.03162157
Nudt7	1600	-0.22	0.06	-3.68	0.00023561	0.03162157
Cecr2	1888	-0.25	0.07	-3.67	0.00024579	0.03253065
Emp2	2136	-0.21	0.06	-3.67	2.46E-04	3.25E-02
Adamts9	2524	-0.24	0.06	-3.66	0.00025421	0.03341257
Irf7	378	0.31	0.08	3.65	0.000259	0.03345537
Acap1	40	0.24	0.07	3.65	2.60E-04	3.35E-02
Mmd	997	-0.26	0.07	-3.66	2.56E-04	3.35E-02
Gm22748	119	-0.30	0.08	-3.65	0.00026156	0.03345537

Pde3a	7039	-0.18	0.05	-3.64	0.00027037	0.03435135
Chrna2	55	-0.27	0.07	-3.64	2.76E-04	3.48E-02
Ptk2b	350	0.29	0.08	3.63	0.00027948	0.03504141
Cd3e	21	0.18	0.05	3.63	0.00028328	0.03528598
Abcg1	267	0.30	0.08	3.62	2.89E-04	3.58E-02
Prex1	783	0.25	0.07	3.60	0.00031475	0.03846596
Plin5	2833	-0.20	0.05	-3.60	3.15E-04	3.85E-02
Rpl3	2683	0.19	0.05	3.60	0.00032269	0.03917132
Clec1b	40	0.23	0.06	3.59	0.00032897	0.03918475
Vegfa	8480	0.17	0.05	3.59	3.27E-04	3.92E-02
Bhlhb9	662	-0.25	0.07	-3.59	0.0003281	0.03918475
Lclat1	4829	-0.19	0.05	-3.59	0.00033283	0.03939832
Ppara	2115	0.20	0.06	3.59	3.36E-04	3.95E-02
mt-Nd4l	73	-0.25	0.07	-3.58	0.00033823	0.03954548
Ammecr1	761	0.25	0.07	3.58	0.00034143	0.03967627
RP23-361M12.2	59	0.27	0.08	3.58	0.00034742	0.04012758
Wipf3	1679	0.23	0.06	3.57	0.00035233	0.04045018
Sbk1	3569	0.21	0.06	3.57	0.0003637	0.04150483
Serpina3g	28	0.17	0.05	3.56	0.00036819	0.04152069
H2-T22	523	0.30	0.08	3.56	0.00036699	0.04152069
Epha4	2924	-0.19	0.05	-3.55	0.00037811	0.04234495
Pirt	448	-0.29	0.08	-3.55	0.00037994	0.04234495
Kcnj2	3162	-0.23	0.06	-3.55	0.00038453	0.04260731
Tmsb4x	6280	0.20	0.06	3.55	0.00038679	0.04260968
RP23-465M17.2	143	0.30	0.08	3.54	0.00040127	0.04369915
H2-T23	716	0.29	0.08	3.54	0.00039912	0.04369915
Serpina3k	9	-0.12	0.03	-3.53	0.00041179	0.0445906
RP23-8J15.5	389	-0.28	0.08	-3.53	0.00042019	0.04524323
Padi4	21	0.16	0.04	3.52	0.00042511	0.04527009
Psmb10	843	0.28	0.08	3.52	0.00042519	0.04527009
Cd84	200	0.30	0.08	3.52	0.00043821	0.04614064
Rasgrp1	51	0.25	0.07	3.52	0.0004382	0.04614064
C1qa	737	0.27	0.08	3.51	0.00045149	0.04701923
Alas1	5818	0.21	0.06	3.51	0.00045044	0.04701923
Gbp7	1010	0.28	0.08	3.50	0.00045768	0.04714825
Myh9	11843	0.18	0.05	3.51	4.56E-04	4.71E-02
Prg4	260	0.30	0.08	3.50	4.70E-04	4.81E-02

Cyp4f18	31	0.22	0.06	3.49	4.78E-04	4.87E-02
Slc2a3	197	0.29	0.08	3.49	4.92E-04	4.93E-02
Lpcat3	904	-0.23	0.07	-3.49	4.91E-04	4.93E-02
Il21r	45	0.25	0.07	3.49	0.00048697	0.04932342
Arid5b	2355	-0.19	0.05	-3.48	4.98E-04	4.97E-02
Ptpn22	65	0.24	0.07	3.48	0.00050314	0.04994185

Supplemental Table S2: Gene-specific primers for qPCR

Gene	Species	Forward primer 5' > 3'	Reverse primer 5' > 3'
Ptgds	Mus musculus	ACTACACCTACAGCAGCCCC	CCTTGGTGCCTCTGCTGAATA
Nr3c2 (MR)	Mus musculus	ATGGGTACCCGGTCCTAGAG	GTTGTGTTGTCCTTCCACGGC
Lcn2 (NGAL)	Mus musculus	GACTTCCGGAGCGATCAGTT	CTGATCCAGTAGCGACAGCC
Lgals3 (Gal3)	Mus musculus	CACTGACGGTGCCCTATGAC	TGAAGCGGGGGTTAAAGTGG
Ikzf3	Mus musculus	GGTGGGAAGATGAACTGCGA	AGATGCCCCGCACTGATTAC
Rasal3	Mus musculus	AGCCTGTGCTTGTGTCAGTG	TCTTGGAGATGAGAGGGGTGT
Cd52	Mus musculus	GCCCAGGAAGATTTCAGGATGA	TTTTGTTAGTACCAGAAGCGGC
Itgb7	Mus musculus	CTGAGTGAGGACTCCAGCAA	TCACCCTCCGTCTTCTCAGG
Grap2	Mus musculus	GCCTCTCTCGACATCAAGCA	GGGAACTTCTCGGTCCACAG
Itgal	Mus musculus	CAGATCTGGACCCCTGCGT	AGACTTGACCCCTCGGTGAG
Hpse	Mus musculus	CTTCGGAGCAGGCAACTACC	GTAACACCCTGGGACCTACC
Amy1	Mus musculus	TTCAGGGACATGGTGAACAGG	CACATGTACTGCTTTGTCCAGC
Mthfd2	Mus musculus	AACGTTGGAATGCCGATTGC	TCTGCAAGGATCGTGTGCTT
Cd37	Mus musculus	ACAGATTACCCTGGGCATCC	CGGTAGCTCTGGATCGTCCT
Thbs1	Mus musculus	AATCATGGCTGACTCGGGAC	GCGCTGGTTATGATTGGCAG
Itgb2	Mus musculus	CGCTAATCCTGAGTTCGACCA	GCCCAGCTTCTTGACGTTGT
Chrna2	Mus musculus	GATGACCACCAATGTCTGGCT	CAAACTCCCCGTCTGCATTG
Pdk4	Mus musculus	GAGCTGTTCTCCCGCTACAG	CGGTCAGGCAGGATGTCAAT
Ucp3	Mus musculus	TGGATGCCTACAGAACCATCG	TCTTGTGATGTTGGGCCAAGT
Angptl4	Mus musculus	GACTGCCAGGAACTCTTCCAA	CAGGCGTCTCTGAATCACTGT
PTGDS	Homo sapiens	GGAAAAACCAGTGTGAGACCC	CAGCGCGTACTGGTCGTAG
18S rRNA	Homo sapiens	AGTCCCTGCCCTTTGTACACA	GATCCGAGGGCCTCACTAAAC
